# Prevalence of anemia and its associated factors among patients with type 2 diabetes mellitus in a referral diabetic clinic in the north of Iran

**DOI:** 10.1186/s12902-023-01306-5

**Published:** 2023-03-09

**Authors:** Reyhane Hizomi Arani, Farima Fakhri, Mohammad Naeimi Tabiee, Fatemeh Talebi, Zahra Talebi, Negin Rashidi, Maryam Zahedi

**Affiliations:** 1 Prevention of Metabolic Disorders Research Center, Research Institute for Endocrine Sciences, Shahid Beheshti University of Medical Sciences, Tehran, Iran; 2 Metabolic Disorders Research Center, Research Institute for Endocrine Sciences, Kerman University of Medical Science, 7616913555 Kerman, Iran; 3 Department of Internal Medicine, Hematology and Oncology disorders, Clinical Research Development Unit (CRDU), Sayad Shirazi Hospital, Golestan University of Medical Sciences, Gorgan, Iran; 4 Clinical Research Development Unit (CRDU), Sayad Shirazi Hospital, Golestan University of Medical Sciences, Gorgan, Iran; 5 Institute of Medical Science, University of Toronto, Toronto, CA Canada; 6 Department of Internal Medicine, Endocrinology and metabolic disorders, Clinical Research Development Unit (CRDU), Sayyad Shirazi Hospital, Golestan University of Medical Sciences, Gorgan, Iran

**Keywords:** Type 2 diabetes mellitus, Anemia, Prevalence, Iran

## Abstract

**Background:**

**Purpose:**

This study intended to investigate the prevalence of anemia and its associated factors among patients with type 2 diabetes mellitus (T2DM) in Gorgan, Iran.

**Methods:**

This cross-sectional study was conducted on 415 (109 men) patients with T2DM referred to the referral diabetes clinic of Sayad Shirazi Hospital in Gorgan in 2021. Demographic information, anthropometric indices, past medical history,  and some laboratory data on cell counts, serum blood glucose, HbA1c, creatinine, lipid/iron profiles, and urinary albumin were collected. The univariable and multivariable logistic regression analysis was applied to compute odds ratios (ORs) and 95% confidence intervals (CI) for potential associated factors, using SPSS version 21. The multivariable Model was adjusted for obesity, Hb A1c, T2DM duration, using glucose-lowering drugs (GLDs), chronic kidney disease (CKD), albuminuria, hypertriglyceridemia, and hypercholesterolemia.

**Results:**

The prevalence of anemia was 21.5% [95%CI: 17.6-25.7] among our total participants. The corresponding values for men and women were 20.2 (13.1-29.0) and 21.9 (17.4-27.0), respectively.The adjusted model revealed that obesity (OR, 1.94 [95% CI, 1.17–3.23]), T2DM duration for more than five years (OR, 3.12 [1.78–5.47]), albuminuria (OR, 6.37 [3.13–10.91]), chronic kidney disease (OR, 4.30 [ 2.83–7.29]) and hypertriglyceridemia (OR, 1.72 [ 1.21–2.77]) were significantly associated with prevalent anemia among patients with T2DM. Moreover, using insulin separately or in combination with oral GLDs associated positively with the prevalence of anemia with ORs of 2.60 [1.42-6.42] and 1.87 [1.30-4.37] , respectively.

**Conclusion:**

Anemia had a high prevalence among patients with T2DM in the north of Iran (about 22%), which is associated with obesity, hypertriglyceridemia, duration of T2DM, and diabetic kidney disease.

## Introduction

Anemia is a condition in which the oxygen-carrying capacity of blood can not meet the physiological needs of the body, due to the decreased erythrocyte mass or hemoglobin concentration [1]. The prevalence of anemia is about 27% worldwide, which makes it a public health problem with the greatest impact on developing countries (they account for more than 89% of the anemia burden) [ 2 ]. Based on global burden disease (GBD) reports, Iran, as a developing country, anemia has the age-standardized prevalence and years lived with disability (YLDs) of 23.0% and 677 (per 100,000), respectively [2], which was more prominent among adult patients with diabetes mellitus (DM) (30.4%) [3].

Nearly 4.5 million cases of DM were detected among Iranian adults in 2011. It is estimated that about 9.2 million of Iranian individuals will be affected by DM by the year 2030. This significant growth in the disease incidence reveals the high burden of DM in Iran, especially when taking into account the impact of its complications [4, 5, 46]. It has been reported that the patients with DM are twice more prone to anemia compared to the individuals without DM [6–8]. Anemia could also increase the risk of end-stage renal and cardiovascular diseases, hospitalization, and premature death in patients with DM [9]. Moreover, it plays a role in the progression and development of macrovascular and microvascular complications of DM [10]. Hence, it could affect the patients’ quality of life and their healthcare costs [11, 46].

Many researchers have studied the potential associated factors with anemia such as age, gender, diabetic nephropathy, glycemic control, antihypertensive medications, and glucose-lowering drugs (GLDs) [12, 13]. Both DM and anemia have some similar symptoms like numbness or coldness in the extremities, pale skin, and shortness of breath. This might result in remaining anemia unrecognized in a considerable number of patients with DM [6]. Therefore, early diagnosis and management of anemia can be recommended as an essential strategy to reduce its adverse effects.

Despite the high prevalence of anemia and its effect on DM complications, in Iran, there are limited studies examining its associated factors. Hence, this study aimed to estimate the prevalence of anemia and its association factors among patients with type 2 diabetes mellitus (T2DM) in a referral diabetes clinic in Gorgan, located in the north of Iran.

## Materials and methods

### Study population

This clinical-based cross-sectional study included outpatients with T2DM being under treatment at the referral diabetes clinic of Sayad Shirazi hospital in Gorgan in 2021. The sample size was calculated based on this formula: N = (pq/e^2^) * z_1−/α_^2^. Here, p was the anticipation of anemia prevalence of 19.6% in the diabetic population, [14] q = 1 - p; e was an allowable error (5%); and Z_1−α/2_= 1.96. So, 242 participants were an acceptable sample size for this study.

To mitigate selection bias, patients were selected by a systematic random sampling technique among volunteer patients with T2DM. In this method, the selection of the first subject is done randomly and then the subsequent subjects are selected by a periodic process.

The exclusion criteria were: (1) age ≤ 18 or ≥ 75 years, (2) T2DM duration less than 1 year (3) known hematologic diseases (thalassemia, lymphoma, and leukemia) or other systemic disorders (such as infectious diseases) that could result in anemia, (4) presence of an acute condition (such as acute bleeding) or hospitalization within the last two weeks before sampling, (5) blood transfusions in the three months before sampling, (6) pregnancy, (7) type 1 DM, (8) smoking, (9) missing clinical and demographic data. Finally, 415 (109 men) eligible participants remained for our analysis.

The Ethics Committee of the Golestan University of Medical Sciences approved this study (ethics Code: IR.GOUMS.REC.1398.170). Verbal informed consent was obtained from all subjects, and they were also assured that their personal information would remain confidential. All methods of this study were performed in accordance with the relevant guidelines and regulations.

### Measurements

A trained interviewer recorded patients’ information, including demographic characteristics, duration of T2DM, and medical history. The height and weight of patients without shoes and with light clothes to the nearest 100 g were measured using the same device (Seca weighing scale, made in Germany), and body mass index (BMI) was calculated by dividing weight (kg) into height squared (meters).

After 8 to 12 h of overnight fasting, a blood sample was taken from all participants to measure cell counts, iron profiles (total iron-binding capacity (TIBC), hemoglobin concentration, serum ferritin, and iron level), triglyceride, high-density lipoprotein cholesterol (HDL-C), low-density lipoprotein cholesterol (LDL-C), total cholesterol (TC), fasting plasma glucose (FPG), and glycated hemoglobin (Hb A1c). Also, urine sample was taken from the participants. All of them were measured in a same lab with same kits, devices, and methods.

FPG was measured by the colorimetric glucose oxidase method (Human, Heidelberg Germany). Furthermore, TC, HDL-C, and triglyceride were measured by enzymatic method using a proper kit (Lipid, Pars Azmoon Co., Karaj, Iran). Also, if triglyceride levels were < 400 mg/dL, the Friedewald formula (LDL = total cholesterol - (HDL + TG/5)) was applied for calculating LDL-C, and if triglycerides were ≥ 400 mg/dL, LDL-C was measured by direct assay. Hb A1C was assessed by column chromatography. TIBC was measured by the chemical precipitation method. Also, ferritin was measured by the immunoassay method using a gamma counter. Urinary albumin excretion was assessed by the immunoturbidometry method of a 24 hour’s urine collection. Serum creatinine levels were measured using kinetic colorimetric Jaffe with a sensitivity of 0.2 mg/dL (range, 0.2–15 mg/dL). The Modification of Diet in Renal Disease (MDRD) equation was employed for calculating the estimated glomerular filtration rate (e-GFR) of the participants [15, 16].

### Definition of outcomes and variables

Anemia was considered as hemoglobin less than 12 g/dL in women and less than 13 g/dL in men according to the World Health Organization (WHO) criteria (12). DM was defined as FPG ≥ 126 mg/dL and/or Hb A1c ≥ 6.5 or taking any GLDs. The participants were categorized into two groups: BMI < 30 kg/m^2^ (normal/overweight) and ≥ 30 kg/m^2^ (obese). Chronic kidney disease (CKD) was considered as e-GFR lower than 60 mL/ min/1.73m^2^ according to the kidney disease outcomes quality initiative (KDQOI) guidelines [17]. Albuminuria was considered as urinary albumin creatinine ratio (ACR) of 30 mg or more in 24 hours’ urine collection. Hypertriglyceridemia was defined as TG more than 150 mg/dL and hypercholesterolemia was considered TC more than 200 mg/dL.

### Data analysis

Statistical analyses were performed using SPSS version 21 for Windows (Chicago, Illinois, USA). To report the quantitative variables, mean (standard deviation: SD) and median (inter-quartile range) were used for variables with normal and highly skewed distribution, respectively. To compare baseline characteristics of quantitative variables between anemic and non-anemic groups, the t-test and the Mann-Whitney test were used for variables with normal and highly skewed distribution, respectively. The prevalence [95% confidence interval (CI)] of each group of categorical variables were estimated and compared by Chi-square test.

The univariable and multivariable logistic regression analyses were performed for categorical variables to evaluate thet association between variables and anemia by reporting odds ratios (ORs) with 95% CI. Only covariates with a p-value <0.20 in the univariable analysis were then selected to enter the multivariable analysis. The multivariable model is adjusted for obesity status, Hb A1c (Hb A1c ≤ 7% as reference), T2DM duration (less than five years as reference), GLDs usage (oral as reference), prevalent CKD, prevalent albuminuria, hypertriglyceridemia, and hypercholesterolemia . 

## Results

The study population consisted of 415 participants with a mean (SD) age of 57.5 (8.6). The prevalence of anemia was 21.5% (95% CI: 17.6-25.7) among our total participants. The corresponding values for our male and female participants were 20.2 (13.1-29.0) and 21.9 (17.4-27.0), respectively. The baseline characteristics of participants are shown in Table 1. Generally, compared to T2DM patients without anemia, T2DM patients with anemia had a longer T2DM duration and higher levels of FPG, HDL-C, and triglycerides. Iron indices (iron, ferritin, and TIBC) were similar among patients with and without anemia, considering this point that we defined anemia based on hemoglobin, and both groups may have negative iron balances. Table 2 shows the prevalence of anemia in different subgroups. Anemia was more prevalent among obese individuals compared to non-obese ones . In addition, the prevalence of anemia was higher in participants with more than five years of T2DM duration than in ones with less than five years. Moreover, participants with Hb A1C > 7% had a higher prevalence of anemia than ones with Hb A1C ≤ 7%. Also, patients with albuminuria and CKD were more likely to have anemia. Furthermore, participants who used a combination of insulin and oral GLDs or used insulin separately were more likely to have anemia compared to those who were treated with oral GLDs only. Finally, participants with hypertriglyceridemia had a higher prevalence of anemia than those with low triglyceride levels.

Multivariable logistic regression analysis (Table 3) showed that the presence of obesity (OR, 1.94 [95%CI, 1.17–3.23]), T2DM duration > 5 years (OR, 3.12 [CI, 1.78–5.47]), albuminuria (OR, 6.37 [CI, 3.13–10.91]), CKD (OR, 4.30 [CI, 2.83-7.29]), as well as hypertriglyceridemia (OR, 1.72 [CI, 1.21–2.77]),had the independent positive associations with anemia. Furthermore, using insulin separately or in combination with oral GLDs associated positively with the prevalence of anemia with ORs of 2.60 [CI, 1.42–5.42] and (OR, 1.87 [CI, 1.30–4.37])), respectively.

## Discussion

In this clinic-based study conducted in 2021, about one-fifth of the Gorgan diabetic women and men were found to have anemia. Obesity, T2DM duration > 5 years, albuminuria, CKD, hypertriglyceridemia, as well as using insulin both with and without oral GLDs were independently associated with the prevalence of anemia among our participants.

Several studies have reported the prevalence of anemia among patients with T2DM to be high, especially among developing countries. Our results were similar to the previous study from Iran (among residents of Tehran city) in 2014 (30.4%) [3]. A recent meta-analysis estimated a prevalence of 35% for anemia among patients with T2DM in Africa [18]. Compared to ours, other studies conducted in Kuwait (6), Malaysia [19], Brazil [20], Greece (among patients with DKD stages 2–4) [21, 22], Saudi [23], England [24], and Pakistan [25], revealed a higher prevalence of anemia among patients with T2DM, which were 29.7% , 31.7%, 34.2% , 34.7%, 47.8%, 55.5%, 59%, and 63%, respectively ; however, a study from India [10] and a community-based cohort from Australia [8] showed a lower prevalence of 12.13% and 11.5%, respectively. These differences may be due to the quality of healthcare services, such as accessibility of patients to visit a specialist and laboratory testing besides. For instance, in developed countries, the participants are in close follow-up in specialized centers, therefore were not representative of the whole diabetic population. Selection bias could also be the underlying cause of the difference;furthermore, the differences could be the result of variations in studies’ methodology and participant characteristics such as lifestyle, feeding habits, type of GLD usage, duration of T2DM, ethnicity, and mean age [21].

The current study showed that T2DM duration > 5 years (regardless of glycemic indices and nephropathy) had a strong independent association with anemia, in line with some other studies [8, 26]. It seems that chronic hyperglycemia could decrease erythropoiesis and increase red blood cells (RBCs) destruction due to more exposure to inflammation and oxidative stress, and bone marrow impairment [27, 28]. In this study, although the levels of FPG and HbA1c were higher among diabetics with anemia, statistically they were not associated with anemia. However, some studies showed lower HbA1c among patients with anemia [29, 30]. They explained that decreased hemoglobin concentration and the enhanced RBC turnover in the anemia of chronic diseases could reduce the glycation process, and consequently lead to falsely reporting lower HbA1c levels [31].

Despite the lack of significant association between glucose indices and the prevalence of anemia, using insulin had a significant association in the present study. Insulin users had potentially worse baseline characteristics rather than non-insulin users; also, using insulin could be a representative of poor control DM or prolonged diabetes or those with complications (allocation bias) [32]. So this result is in agreement with some other studies reporting that poor control DM has an association with anemia [12, 33]. Considering that neuropathy is common in patients with poor glycemic control, one of the reasons for the increased risk of anemia is impairment of production and release of the erythropoietin stimulated by the autonomic nervous system [34]. Furthermore, DM could negatively affect the interstitial and peritubular structures of kidney (where erythropoietin is produced), and anemia could be the result of decreased erythropoietin production by kidney failure. Moreover, the exposure of erythroblasts or mature erythrocytes to oxidative stress (due to glucose toxicity) could cause erythrocyte dysfunction. Besides, metformin is the first-line choice for T2DM management unless in patients with e-GFR < 45 ml/min/1.73 m^2^, according to ADA 2022 guidelines. It has been reported that metformin interferes with Cyanocobalamin absorption and is associated with vitamin B12 deficiency, resulting in an increased risk of anemia among patients with T2DM [35]. 

We found that obesity (BMI ≥ 30 kg/m^2^) and hypertriglyceridemia(triglycerides> 150 mg/dL) were independently associated with the prevalence of anemia after adjustment with other confounders. This could be explained by the hypothesis that the adipose tissue is the source of different cytokines. The increase in inflammatory activity of adipose tissue in obese patients could cause an increase in hepcidin levels, leading to a reduction in serum iron [36]. However, some studies reported an “obesity paradox” in anemia; they observed that normal/overweight T2DM patients were more anemic than obese patients [29].They also claimed that overnutrition may be associated with increased consumption of protein, iron, and other micronutrients, which have a protective effect against iron and/ or B12 deficiency in the diabetic population [37].

We found that albuminuria > 30 mg/24hr and e-GFR≤60 ml/min/1.73 m^2^, as two important parameters of diabetic kidney disease (DKD), had a significant independent association with anemia among patients with T2DM. Our findings were in accordance with other studies showing a higher frequency of anemia in patients with T2DM and nephropathy [38, 39]. A large multicenter US study of the Kidney Early Evaluation Program (KEEP) demonstrated that the development of anemia in patients with DKD had a statistical relationship with the severity of albuminuria and e-GFR decline [40]. DM could also damage tubulointerstitial tissues (associated with the degree of albuminuria) in the early stage, even before any reduction in e-GFR. It could cause decreased erythropoietin production and iron metabolism impairment leading to reduced production of RBCs [41]. Blood urea may be increased due to renal dysfunction, and it could negatively affect RBC’s lifespan [22].

We found no significant sex-difference in the prevalence of anemia, in contrast to other studies [12, 42]. As the mean age of our participants was 57.8 years, men and women were similar due to decreased occurrence of anemia in women due to menopause. Moreover, in the Iranian diabetic society, men and women have identical diets, education, and health awareness [43].

The present study has several strengths. First, we assessed various baseline characteristics, such as different laboratory and clinical data. We considered different potential associated factors in our model in a sufficient sample size. Thus, we could discuss some important independent associated factors in detail. Second, we randomly selected our participants out of a referral center in Gorgan that could enhance the ability to generalize our results to other Iranian populations (due to different ethnic groups living in Gorgan, including indigenous inhabitants, Turkman, Sistani, and Baloch). Finally, to the best of our knowledge, it is the first report on the prevalence of anemia among a diabetic population in the north of Iran.

This research had several limitations. First, as it was a cross-sectional study, we could not show the casualty, so a longitudinal study is needed to assess the relationship over time. Second, we did not consider some drug usage including iron supplements and erythropoietin in the case of CKD. Third, we did not record dietary patterns, particularly iron intake was not considered in our study, although all patients were routinely educated about diet and health care in our referral diabetes clinic. Fourth, we did not measure erythropoietin, B12, and folate levels in our participants, so the lack of definition of anemia etiology was another limitation. Fifth, we did not exclude patients with high blood pressure [39] and patients who have occupations leading to anemia (for example occupations contacting with blood toxins, such as lead, benzol, amino- nitro compounds of benzol, arsenic hydrogen, etc.) [44, 45]. Finally, considering only one center for this study was the other part of our limitations.

In conclusion, we demonstrated a high prevalence of anemia among patients with T2DM in one of the referral diabetic clinic in the north of Iran, which was associated with obesity, hypertriglyceridemia, duration of T2DM, and renal dysfunction. It is necessary to screen and evaluate anemic conditions in diabetic populations. They must be warned against the potential risk and complications of anemia and the importance of regular screening, especially among those with stated associated factors.

**Table 1:** Baseline characteristics of 415 participants with T2DM

	Total (N = 415)	Without Anemia (N = 89)	With Anemia (N = 326)	P-value
Age (year)	57.8 (9.0)	57.4(8.5)	57.9(9.1)	0.742
BMI (kg/m^2^)	28.4 (4.0)	28.2(4.2)	28.3(4.0)	0.939
Diabetes duration (month)	10.7 (6.2)	7.2(4.8)	11.1(6.6)	**< 0.001**
FPG (mg/dL)	180.1 (141–244)	162.2(129-198)	185.0(145-257)	**< 0.001**
Hb A1c (%)	8.1 (8.9)	8.5(1.8)	8.9(1.7)	0.176
TC (mg/dL)	175.7 (3.2)	165.4 (0.7)	198.5 (3.9)	0.073
LDL-C(mg/dL)	76.8 (59–103)	83.5(67-103)	75.0(58-103)	0.108
HDL-C(mg/dL)	48.4 (43–64)	46.0(38-55)	49.0(45-67)	**0.009**
Triglyceride(mg/dL)	158.9 (106–225)	140 (101–182)	164.0(108-237)	**0.014**
e-GFR (ml/min/1.73 m^2^)	82.1 (3.5)	86.3(2.5)	80.9(3.8)	0.202
Hemoglobin(g/dL)	12.0 (1.4)	13.2(1.6)	11.7(1.4)	**< 0.001**
Ferritin (µg/L)	37.1 (21–72)	34.0(27-65)	37.9(20-74)	0.740
Iron (µg/dL)	82.1 (59–98)	79.0(65-98)	83.0(57-98)	0.254
TIBC (µg/dL)	351.7 (36.2)	347.1(33.0)	352.9(37.1)	0.292

Data were presented as mean (SD); FPG, LDL-C, HDL-C, Triglyceride, Ferritin and Iron were presented as median (interquartile range [IQR]); For comparison between the two groups, t-test for data with normal distribution and Mann-Whitney test ones with abnormal distribution; BMI: body mass index; FPG: fasting plasma glucose; Hb A1c: Hemoglobin A1c; TC: total cholesterol; LDL-C: low density lipoprotein cholesterol; HDL-C: high density lipoprotein cholesterol; e-GFR: estimated glomerular filtration rate e-GFR: estimated glomerular filtration rate; TIBC: total iron binding capacity; T2DM: Type 2 diabetes mellitus

**Table 2:** Prevalence of anemia among patients with T2DM in different subgroup

	Case/Total	Crude prevalence % (95% CI)	P-value
**Obesity**			**0.032**
Non-obese	53/292	18.2 (13.9–23.1)	
Obese	36/132	27.3(19.9–35.7)	
**Gender**			0.708
Women	67/306	21.9(17.4–27.0)	
Men	22/109	20.2(13.1–29.0)	
**Diabetes Duration**			**<0.001**
< 5 years	18/162	11.1(6.7–17.0)	
> 5 years	71/253	28.1(22.6–34.0)	
**Hb A1c**			**0.001**
≥ 7	11/106	10.4(5.3–17.8)	
> 7	78/309	25.2(20.5–30.5)	
**Albuminuria**			**<0.001**
No	64/375	17.1(13.4–21.3)	
Yes	25/40	62.5(45.8-77.3)	
**GLD**			**<0.001**
Oral	52/300	17.3(13.2–22.1)	
Insulin	24/77	31.2(21.1–42.7)	
Oral + Insulin	13/38	34.2(19.6–51.4)	
**CKD**			**<0.001**
No	62/362	17.1 (13.4–21.4)	
Yes	27/53	50.9(36.8-64.9)	
**Triglyceride**			**0.023**
≤ 150	39/226	17.3 (12.6–22.8)	
> 150	50/189	26.5 (20.3–33.4)	
**TC**			0.066
≤ 200	67/340	19.7(15.6–24.3)	
> 200	22/75	29.3 (19.4–41.0)	
**LDL-C**			0.688
≤ 100	65/296	22.0(17.4–27.1)	
> 100	24/119	20.2 (13.4–28.5)	
**HDL-C**			0.240
≤ 40	63/272	23.2 (18.3–28.6)	
> 40	26/143	18.2(12.2-25.5)	

Each group of categorical variables compared by Chi-square test; GLD: glucose lowering drug; CKD: chronic kidney disease; TC: total cholesterol; LDL-C: low density lipoprotein cholesterol; HDL-C: high density lipoprotein cholesterol; T2DM: Type 2 diabetes mellitus

**Table 3:** Crude and adjusted odds ratios of associated factors with prevalent anemia among patients with T2DM

	Crude OR (95% CI)	Adjusted OR (95% CI)	P-value
Obesity			
Non-obese	1	1	
obese	2.28 (1.72–3.19)	1.94 (1.17–3.23)	**0.010**
**Hb A1c**			
>7	1	1	
≤7	1.61 (1.31–2.12)	1.15 (0.60–3.10)	0.610
**Diabetes Duration**			
<5 years	1	1	
≥ 5 years	4.23 (2.16–6.01)	3.12 (1.78–5.47)	**<0.001**
**Albuminuria**			
No	1	1	
Yes	7.31 (4.61–12.54)	6.37 (3.13–10.91)	**<0.001**
**GLD**			
Oral	1	1	
Insulin	2.42 (1.89–5.17)	1.87 (1.30–4.37)	**0.039**
Oral + Insulin	3.19 (2.03–6.73)	2.60 (1.42–5.42)	**0.046**
**CKD**			
No	1	1	
Yes	5.87 (3.51–8.15)	4.30 (2.83–7.29)	**<0.001**
**Triglyceride**			
≤ 150	1	1	
> 150	2.31 (2.79–3.98)	1.72 (1.21–2.77)	**0.024**
**TC**			
≤200	1	1	
> 200	2.09 (0.67–5.45)	1.58 (0.84–4.88)	0.124

Multivariable logistic regression analysis was performed in 2 levels: (1) without adjustment (crude odds ratios (ORs) and 95% CI); (2) full adjustment, which is adjusted for obesity, Hb A1c, diabetes duration, GLDs usage, CKD, albuminuria, triglyceride and TC. GLD: glucose lowering drug; CKD: Chronic kidney disease; TC: total cholesterol; LDL-C: low density lipoprotein cholesterol; HDL-C: high density lipoprotein cholesterol; T2DM: Type 2 diabetes mellitus

## Data Availability

The data that support the findings of this study are available from Golestan University of Medical Sciences but restrictions apply to the availability of these data, which were used under license for the current study, and so are not publicly available. Data are however available from the authors upon reasonable request and with permission of Golestan University of Medical Sciences.

## List of abbreviations

GBD: Global burden disease
YLDs: Years lived with disability
T2DM: Type 2 diabetes mellitus
DM: Diabetes mellitus
GLDs: Glucose-lowering drugs
BMI: Body mass index
TIBC: Total iron-binding capacity
HDL-C: High-density lipoprotein cholesterol
LDL-C: Low-density lipoprotein cholesterol
TC: Total cholesterol
FPG: Fasting plasma glucose
HbA1c: Glycated hemoglobin
MDRD: Modification of Diet in Renal Disease
e-GFR: Estimated glomerular filtration rate
WHO: World Health Organization
DKD: Diabetic kidney disease
KDQOI: Kidney disease outcomes quality initiative
ACR: Albumin creatinine ratio
CI: Confidence interval
ORs: Odds ratios
RBCs: Increase red blood cells
KEEP: Kidney early evaluation program
EPO: Erythropoietin.
